# Effect of different shading materials on grain yield and quality of rice

**DOI:** 10.1038/s41598-019-46437-9

**Published:** 2019-07-10

**Authors:** Hong Chen, Qiu-Ping Li, Yu-Ling Zeng, Fei Deng, Wan-Jun Ren

**Affiliations:** 1 Key Laboratory of Crop Ecophysiology and Farming System in Southwest China of Ministry of Agriculture, Crop Ecophysiology and Cultivation Key Laboratory of Sichuan Province, Sichuan Agricultural University, Chengdu, China; 2 Institute for New Rural Development, Sichuan Agricultural University, Ya’an, China

**Keywords:** Abiotic, Light responses

## Abstract

Light is a basic environmental factor required for plant growth and development; however, these are not only affected by light quantity, but also by light quality - light and radiation of different wavelengths and different compositions. In four different rice varieties (*Oryza sativa* L.), two kinds of shading materials, white cotton yarn (Shading (W)) and black nylon net (Shading (B)) were used to simulate cloudy days. Yield decreased under Shading (W) (15.3–17.7%) and Shading (B) (16.6–20.0%) compared to under sunny day (CK), and different effects on rice quality, which is mainly affected by changes in light quality, were observed. The change in light quality (Blue, Green, Red and R/FR proportions) represented under Shading (W) was significantly different from that under CK and Shading (B) conditions. Red light composition under Shading (W) was closer to that of the cloudy day condition. The proportion of blue light under Shading (W) was significantly lower than that under CK conditions; under Shading (B), it was higher than that under all conditions. The differences in light quality may affect photosynthesis in leaves and final starch synthesis, resulting in increased chalky grain rate, chalkiness, and poor rice quality. White cotton yarn as the shading material for further research used to simulate the influence of the light environment on rice growth under cloudy conditions will be better than black net.

## Introduction

Light, temperature, moisture, etc. are all important factors affecting the growth process of plants. Light is not only a major participant in plant photosynthesis, but also affects the relative content and quality of various macromolecules in plants through the formation and transport of photosynthetic products^1–3^.

However, at present, cloudy and rainy weather and increasing number of haze events have aroused people’s concern; In addition, the net amount of radiation reaching the earth’s surface is falling^4^). Sichuan is the largest industrial province in southwest China, there is a serious risk of air pollution that could mask or reflect radiation reaching the plant. With the improvement of the original radiation environment, shading has become one of the main factors restricting the production of rice and other crops in Sichuan^5,6^.

Several studies have shown that in shading experiments at different growth periods of crops, shading after grain filling at the seed bearing stage has the greatest impact on grain filling condition, quality formation, and yield^5,7,8^. After shading, the soluble sugar, sucrose, starch, chalkiness, and yield changed to different degrees, resulting in poor enrichment and poor quality^9^.

Many reports have different conclusions about the effects of weak light on plant growth^9–12^. This is closely related to the test materials selected by researchers, the shading intensity set, the shading period, the number of shading days, and the local ecological environment. Shading not only causes changes in light intensity, but also causes changes in environmental factors such as light quality, air humidity, CO_2_ concentration, and soil temperature^13,14^.

In addition to the intensity and duration of light, different compositions of light matter; blue, red, and far-red light play a key role in controlling plant photomorphogenesis. Changes in light quality affect crop biomass reduction^15^. Red light inhibits internode elongation, promotes lateral branching and tillering, and delays flower differentiation, which has positive effects on resistance to abiotic stresses^16,17^. Different red/far-red (R/FR) ratios can also alter salt and temperature resistance in plants^18^,^19^ and be responsible of intermediate coleptile length and rootlet number in rice^20^. The research on the effect of light quality on rice quality needs further research.

Nowadays, the study of the effects of weak light on rice growth is mostly based on natural disasters which have occurred frequently in recent years. Researchers basically use black nylon nets for shading. There is still a gap between the use of black nylon nets to simulate shading and the accurate simulation of rainy and cloudy weather and the natural environment. Whether the obtained test results can accurately reflect the problems encountered in production practice remains to be further verified.

In this study, the effects of different light conditions on the grain filling and yield of rice under different light environments were analyzed using different materials to cover the shading period of rice during the grain filling stage, so as to further provide a feasible test method for crop growth and quality formation under weak light conditions.

## Results

### Environment of rice canopy

**Figure 1:**
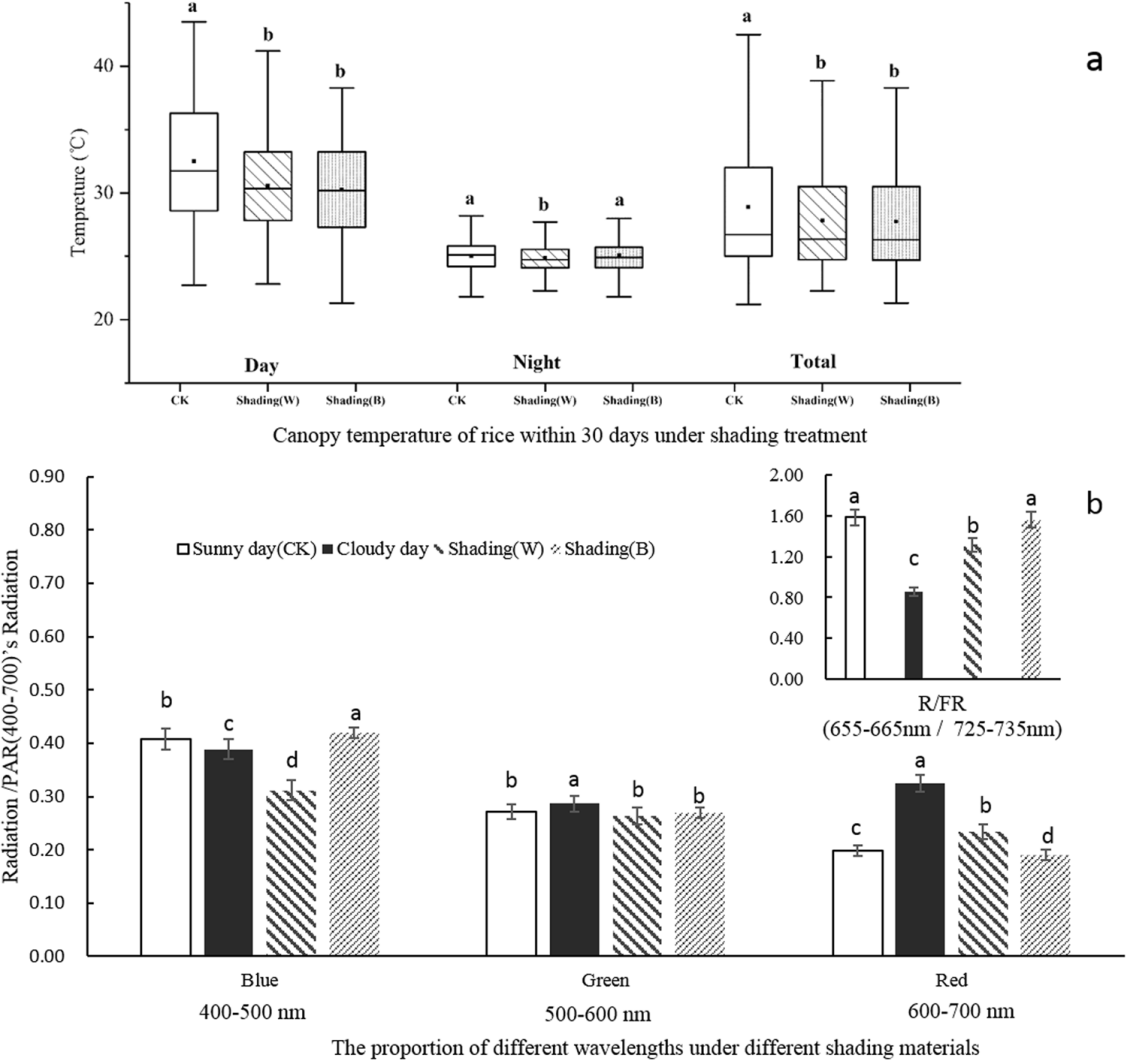
Climatic conditions of rice canopy under different shade materials (Wenjiang, 2018). (**a**) Canopy temperature of rice within 30 days under shading treatment. The top and bottom line segments represent the maximum and minimum values of the data, respectively. Where the upper and lower line segments of the box plot represent the third quartile and the first quartile respectively. The bold lines in the middle of the box plot represent the median of the data, and the black squares represent the average of the data; (**B**) wavelength composition under different shading materials. Different lower-case letters within the column indicate statistically significant differences between treatments for the same variety at the 5% significance level.

As shown in Fig. 1, the temperature of the rice canopy showed different changes between control (CK) and treatments (Fig. 1a). The average daily temperature under the three different light environments, CK, Shading (W), and Shading (B), was 28.90 °C, 27.81 °C, and 27.75 °C, respectively. The average daytime temperature in the control group was 32.51 °C. The average temperature of the control during daytime and the whole day were significantly higher than that of Shading (W) (1.9 °C and 1.1 °C, respectively) and Shading (B) (2.2 °C and 1.2 °C, respectively). Temperature of Shading (W) at night was significantly lower than that of the control and Shading (B).

The temperature difference between daytime and nighttime under different light conditions is 7.5 °C, 5.7 °C, and 5.2 °C, respectively. Compared with the control, the accumulated temperature was decreased by 33.63, 35.60 °C, respectively, under the Shading (W) and Shading (B), during the entire study period.

The radiation of each wavelength was less under Shading (B) than that under control and Shading (W), and effective photosynthesis radiation (PAR) of Shading (B) was only 32% that of the control, while that of Shading (W) was 83%. From the composition of light quality, Shading (W) was more similar to the light quality of cloudy days (Fig. 1b). The R/FR value was significantly lower under cloudy day than under the control (*p* < 0.05); under Shading (W) it was slightly less than that under the control and closer to that of cloudy days than Shading (B). The composition of blue and red light under shade was significantly different from that under cloudy and sunny days. Red light under Shading (W) was closer to that under cloudy days, although blue light under Shading (B) was more similar than Shasing (W) to these conditions. The cloudy day caused the rice crop to absorb blue and red light, and the radiation energy in the two bands was greatly reduced.

### Chlorophyll content

**Figure 2:**
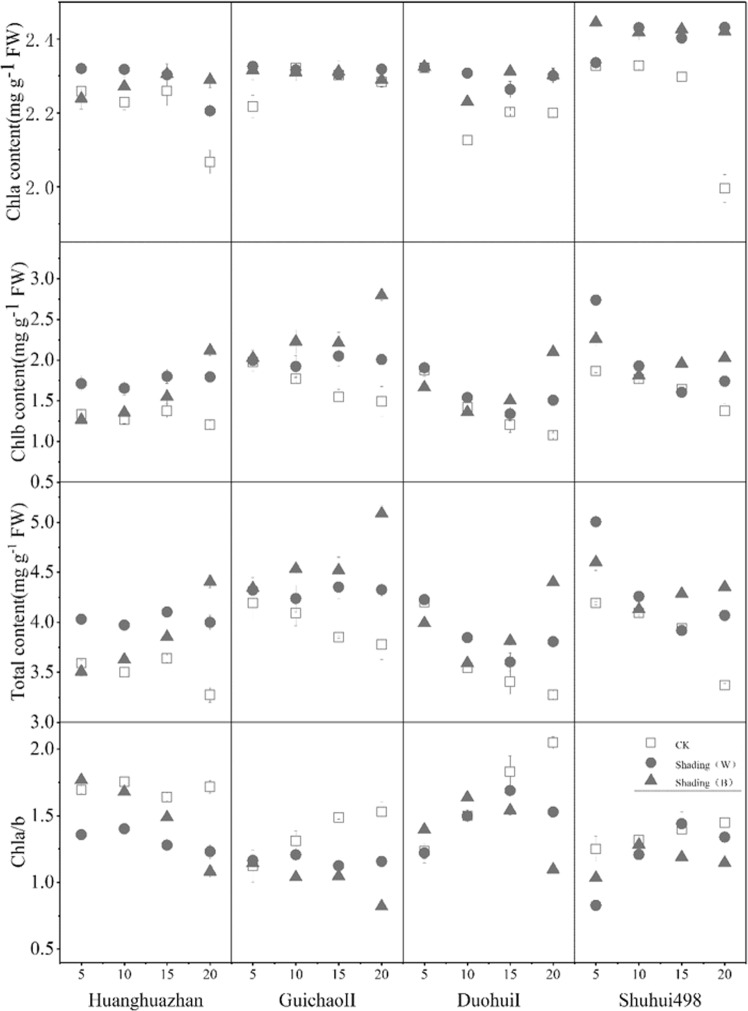
Effect of shading on content of Chl (chlorophyll) a, Chl b, and total Chl, and Chl a/b of rice at 5, 10, 15, and 20 d after heading.

Shading stress, compared to that with the CK, markedly increased the total chlorophyll (Chl) content in the flag leaf by improving both the Chla and Chlb contents, while decreasing Chl a/b (Fig. 2). The change in Chlb content may be the main reason. The Chla content increased under shading, while a gradual reduction was observed in the CK; however, the responses of the Chl a/b, Chla, and Chlb contents were different among the rice varieties (Fig. 2). The Chlb and total Chl contents of Duohui I and Shuhui498 initially decreased and later had a small increase under shading, while those in Huanghuazhan and Guichao II continued increasing. The change in Chl a/b levels to shading was opposite that of the change in Chlb content among the rice varieties (Fig. 2). The Chlb and total Chl content of Duohui I and Shuhui498 initially increased and later had a small decrease under shading, while Huanghuazhan and Guichao II continued decreasing. Each of these turning points occurred at 15 d after shading.

Different varieties have different performances under different light treatments. From the beginning of shading, Chl content (Chlb and total Chl) in Huanghuazhan under Shading (W) was higher than that under Shading (B), but the difference between the two gradually reduced with time, until the Chl content in Shading (W) was lower than that under Shading (B) at 20 d. The turning point for Shuhui498 and Duohui I appeared at 15 d. The Chl content (Chlb and total Chl) in Guichao II was always higher under Shading (B) than under Shading (W). This suggests that different genotypes of rice show different performance under different light treatments.

### Chlorophyll fluorescence parameters

Different varieties of rice Y(II) (actual photochemical efficiency) showed different performance under light treatment (Table 1). Fv/Fm (original light energy conversion efficiency) parameter was in the normal range of 0.80–0.85 in all treatments studied in all varieties, being and significantly lower under CK conditions compared to both Shading (W) and Shading (B) (Table 1). Furthermore, under different shading stress, different treatments showed different increases in 1-qP (photochemical quenching) values, which were significantly lower than those in CK (P < 0.05, except for Huanghuazhan under Shading (B)) (Table 1). These results suggest these varieties have the better ability to capture electrons for PSII reactions under shading stress.

**Table 1:** Chlorophyll fluorescence parameters in rice flag leaves at grain filling stage under shading conditions (Wenjiang, 2018).

Variety	Treatment	Y(II)	^F^v^/F^m	1-qP	NPQ	Y(NO)	Y(NPQ)
Huanghuazhan	CK	0.522a	0.798b	0.267a	0.315c	0.471a	0.008c
	Shading(W)	0.447b	0.841a	0.399b	0.823a	0.307b	0.255a
	Shading(B)	0.514a	0.843a	0.334a	0.604b	0.309b	0.189b
	CK	0.506a	0.799a	0.235a	0.285b	0.387a	0.108c
Guichao II	Shading(W)	0.439b	0.842b	0.414c	0.773a	0.317b	0.245a
	Shading(B)	0.529a	0.823b	0.325b	0.439b	0.333b	0.149b
	CK	0.514a	0.800b	0.295a	0.310c	0.372a	0.115b
Duohui I	Shading(W)	0.515a	0.839a	0.320b	0.631a	0.301b	0.188a
	Shading(B)	0.522a	0.824a	0.328b	0.469b	0.327b	0.158a
	CK	0.545a	0.789b	0.246a	0.451b	0.318a	0.143b
Shuhui498	Shading(W)	0.453b	0.838a	0.387c	0.784a	0.312a	0.242a
	Shading(B)	0.539a	0.839a	0.308b	0.471b	0.319a	0.149b
	V	78.0**	41.7**	143.1	188.7**	79.2**	192.5*
*F*-value	L	7.9**	0.519	2.6**	9.4**	13.0**	4.8**
	V*L	9.1**	1.1	11.6**	5.1*	17.2**	21.4**

Note: Fv/Fm, original light energy conversion efficiency; Y(II), actual photochemical efficiency; qP, photochemical quenching; NPQ, non-photochemical quenching; Y(NO), non-regulated losses of excitation energy including heat dissipation and fluorescence emission; Y(NPQ), regulated energy losses of excitation energy by heat dissipation involving ΔpH- and zeaxanthin-dependent mechanisms. CK (sunny day), Shading(W) (white cotton yarn) and Shading (B) (black nylon net). According to the Fisher’s test, different lowercase letters within the column indicate statistically significant differences between treatments for the same variety at the 5% significance level. L, light intensity and V, variety. *P < 0.05, **P < 0.01.

Under shading stress, the NPQ (non-photochemical quenching) value was significantly higher than that under CK (P < 0.05) (Table 1). The NPQ values of all varieties under Shading (W) were higher than those under Shading (B), indicating that plants can defend against adverse conditions such as light inhibition by releasing more heat energy, and play the role of self- protectors of photosynthesizers (Table 1).

### Yield and yield components

The analysis of yield showed that varieties had significant influence on yield and each component factor, and light had significant influence on spikelet filling, 1000-grain weight and yield. Shading led to a significant reduction in 1000- grain weight and spikelet filling, which ultimately led to a reduction in yield of 15.3% to 20.0% (Table 2). The reduction under Shading (B) was higher than that under Shading (W). Among them, Guichao II had the smallest decrease in Shading (W), only 15.3%. Shuhui498 had the biggest decrease in Shading (B), 20.0%. Furthermore, the shading tolerances of rice varieties were different. Grain weight (except for Duohui I) was significantly (*P* < 0.05) reduced with shading. Shading significantly (*P* < 0.05) reduced spikelet filling and grain weight. There was an 8.3 to 20.7% decrease in spikelet filling caused by shading during the 30 d after heading. Among them, Duohui I had a rate of decline of more than 20%, and Guichao II had the lowest rate of only 8.3%. Duohui I had a small decrease in grain weight, but because the seed setting rate decreases too much, the yield reduces greatly, indicating that the seeding rate under shading is an important factor in the reduction of production. Guichao II had the minimum decrement of grain weight and spikelet filling, which resulted in a smaller reduction in grain yield. This might suggest a higher ability to recover from shading stress.

**Table 2:** Effect of shading on yield and yield components of rice (Wenjiang, 2018).

Variety	Treatment	Grain yield (t/hm^2^)	1000-grain weight (mg)	Spikelet filling (%)
Huanghuazhan	CK	8.50a	25.47a	96.54a
	Shading(W)	7.14b	23.65b	87.16b
	Shading(B)	7.09b	22.87b	85.27b
	CK	8.56a	29.70a	92.09a
Guichao II	Shading(W)	7.25b	28.33b	84.44b
	Shading(B)	7.05b	27.19b	82.51b
	CK	6.33a	32.17a	90.26a
Duohui I	Shading(W)	5.21b	31.12a	71.55b
	Shading(B)	5.14b	31.68a	73.21b
	CK	7.91a	37.12a	94.48a
Shuhui498	Shading(W)	6.56b	34.51b	82.17b
	Shading(B)	6.33b	34.37b	81.77b
	V	13.9**	365.0**	24.9**
F-value	L	14.1**	26.1**	73.0**
	V*L	0.4	1.91	2.2

Note: CK (sunny day), Shading(W) (white cotton yarn) and Shading (B) (black nylon net). Different lowercase letters within the column indicate statistically significant differences between treatments for same variety at the 5% significance level. L, light intensity and V, variety. *P < 0.05, **P < 0.01.

### Amylose content and appearance quality in grains

The varieties had a significant effect on the content of amylose and the appearance quality, while light significantly affected the chalkiness rate and chalkiness score, as well as the content of amylose at 30 days. The content of amylose in the mature stage was significantly decreased under shading treatments compared to that in CK, and the rate of decrease under Shading (B) was lower than that under Shading (W) (Table 3). The chalkiness and chalkiness rate under both treatments were significantly higher than in CK, (except for Guichao II; its chalkiness rate was lower under treatments than in CK, and the quality became better, which may be related to Guichao II itself being a high chalkiness variety and the formation process of chalkiness), and the amplification under Shading (B) was significantly lower than under Shading (W) (Table 3). Therefore, the amylose content of the grain under Shading (B) was higher than under Shading (W) and the chalkiness rate and the chalkiness were lower under Shading (B) than those under the Shading (W). Grain with better appearance quality than under the Shading (W) was obtained.

**Table 3:** Effect of shading on amylose content and appearance quality of rice grains (Wenjiang, 2018).

Variety	Treatment	Amylose Content (%)				Chalkiness	Chalkiness
		15 d	20 d	30 d	mature	rate (%)	score (%)
Huanghuazhan	CK	18.48a	16.8a	18.03a	16.94a	5.25b	1.25a
	Shading (W)	18.28a	16.04a	16.31b	14.98a	11.67a	4.57a
	Shading (B)	16.19a	16.46a	17.14ab	15.1a	7.77b	2.07a
	CK	19.87a	24.44a	27.12a	24.68a	88.97a	25.37a
Guichao II	Shading (W)	17.38a	24.33a	19.61c	17.76b	85.85a	23.90a
	Shading (B)	17.84a	19.27a	21.76b	23.25a	84.57a	27.80a
	CK	14.28a	15.13a	18.5a	14.01a	4.27b	1.07a
Duohui I	Shading (W)	14.13a	14.79a	13.95b	13.58a	15.27a	4.73a
	Shading (B)	14.47a	17.09a	13.16b	13.92a	6.50b	1.83a
	CK	14.52a	16.86a	16.07a	16.43a	8.00b	2.33b
Shuhui498	Shading (W)	15.05a	15.92a	15.18a	12.35b	22.87a	7.10a
	Shading (B)	14.89a	15.88a	14.67a	15.21ab	18.23a	6.20a
	V	27.4**	20.1**	121.6**	22.4**	589.4**	201.36**
F-value	L	2.0	0.8	50.8**	3.4	8.3**	4.2*
	V*L	1.9	1.9	8.8**	2.4	2.4	1.42

Note: CK (sunny day), Shading(W) (white cotton yarn) and Shading (B) (black nylon net). Different lowercase letters within the column indicate statistically significant differences between treatments for same variety at the 5% significance level. L, light intensity and V, variety. *P < 0.05, **P < 0.01.

In the process of grain filling, the content percentage of amylose first increased and then decreased. It may be related to the reason that amylose was synthesized more in the early stage of grain filling and amylopectin was synthesized more in the middle and late stages of grain filling.

### Correlation analysis of light quality, quality and yield of rice

The results of correlation analysis showed that there were no significant correlations between the change in light quality and the quality and yield of rice after ripening. Further analysis showed that the appearance quality of high chalky rice variety (Guichao II) was opposite to that of the low chalky rice varieties (Huanghuazhan, Duohui I, and Shuhui498) under different light conditions (Table 4). The components of R/FR in the light quality of low chalky varieties were significantly correlated with chalkiness degree and chalky grain rate, while only green was significantly correlated with amylose content in the high chalky variety. This may be caused by differences in the formation process of chalky rice in different genotypes.

**Table 4:** Correlation analysis of light quality, grain quality and yield among different rice varieties.

Variety	Light quality	Amylose Content	Chalkiness rate	Chalkiness score	Grain yield
All	Blue	0.345	−0.081	−0.067	0.199
	Green	0.380	−0.090	−0.093	0.382
	Red	−0.333	0.079	0.061	0.350
	R/FR	0.369	−0.088	−0.083	−0.573
Low chalkiness (Huanghuazhan, Duohui I, Shuhui498)	Blue	0.535	−0.606	−0.633	0.203
	Green	0.630	−0.706	−0.737	0.382
	Red	−0.510	0.579	0.605	−0.167
	R/FR	0.596	−0.670*	−0.700*	0.305
High chalkiness (Guichao II)	Blue	0.955	0.134	0.846	0.294
	Green	0.999*	0.462	0.616	0.601
	Red	−0.935	−0.071	−0.878	−0.234
	R/FR	0.994	0.318	0.731	0.469

*Represents a significant correlation between the two factors, *P* < 0.05.

## Discussion

### Environmental impact of different shading materials

The analysis of light quality considers that blue light has a short wavelength and is easily scattered, while red light mainly comes from direct sunlight. It has been reported that the black nylon net commonly used in low light research affects the light quality, especially the R/FR light ratio^21^. The green shade shelter not only reduces the R/FR ratio, but also reduces the proportion of blue light^10^. The proportion of R and FR light decreased after the use of the shade net, and the proportion of blue light increased significantly.

After shading, the direct light source is blocked, thus, the scattered blue light is concentrated in the shade^22^. Another study^23^ used a colorless transparent plastic mesh cover to simulate cloudy treatment, and found that the radiant energy of blue and red light and the ratio of total radiant energy in the absorption spectrum of the population decreased. However, there is also a report that the shading material cannot change the spectral composition and only reduces the light intensity^24^. In our study, the composition of canopy light environment after Shading (W) treatment was significantly lower in blue light than CK, and significantly higher in red light than CK, which was closer to cloudy environment. The composition of blue light and red light in the light environment under the Shading (B) treatment is closer to CK, in which blue light is significantly higher than CK and red light is significantly lower than CK.

Yamazaki^12^ believes that although the black nylon net does not change the R/FR light ratio, it cannot simulate the shade of natural trees in many shading experiments. That’s the same thing as our result. In future studies, attention should be paid to the changes in the spectral composition of the rainy and weak weather compared to the sunny days.

### Effects of different shading materials on photosynthetic characteristics

The chlorophyll content of plant leaves grown under blue light is lower than that under red and white light^25^. The chlorophyll synthesis and chloroplast formation of higher plants and the chloroplasts with high chlorophyll a/b ratio require blue light^26^.

In our research, compared to Shading (B), chlorophyll content increased more 5 d after Shading (W) treatment under low light, resulting in lower photosynthetic efficiency (Y(II)) and more heat dissipation (NPQ), which reduced its light energy utilization rate. These results suggest that plants are more sensitive to stress in low R/FR light environment.

Some research has shown that the proportion and composition of red, blue, and red/far-, infra- red light in a light environment play a very important role in plant growth^27^. The reduction of R/FR under the canopy may cause a decrease in plant photosynthesis, leaf area reduction, plant height increase, biomass allocation pattern change, etc^28,29^. However, the chlorophyll content and the photosynthetic rate per unit leaf area of birch tissue culture seedlings grown under blue light were the highest, while those of the seedlings growing under red light were the lowest^27^. The composition ratio of blue light in Shading (B) slightly increased (2.99%), and the proportion of blue light in Shading (W) decreased (23.58%) compared to that in the control. At the same time, the composition ratio of red light decreased in Shading (B) (3.92%) and increased in Shading (W) (18.37%). Compared with CK, the proportion of R/FR under Shading (B) showed no significant change, and the chlorophyll content increased after blue light increased significantly. Although the proportion of blue light was decreased in Shading (W) treatment, the serious decrease of R/FR ratio also increased the chlorophyll content. This indicates that the composition of F/FR and blue light play a very important role in plant growth.

The Chlb content increased and Chl a/b decreasedmore under Shading (B) than under the Shading (W) at 20 d after shading; PSII light acquisition antenna size and PSII:PSI content changes are usually affected by the Chl a/b ratio. This inference is strengthened by our findings on Chl fluorescence (Table 1). The qP (photochemical quenching) and Y(II) of the tested samples under Shading (B) were higher than those under Shading (W) which may indicate non-radiative (thermal) energy dissipation. Non-photochemical quenching (NPQ) is a non-photochemical quenching coefficient, which refers to the ability of plants to dissipate excess heat energy to defend against the destruction of photoinhibition, etc., and plays a role of self-protection for photosynthetic mechanism (i.e. photosynthesis). And the reduction in NPQ is associated with decreases in non-photochemical quenching. In plants, the response of photosynthesis to the change of light quality may be regulated by the change of PSII activity. We found that the change rules of Y(II) and qP were inhibited. Blue light promoted the Y(II) and qP in leaves, which was consistent with the results of Wang *et al*.^25^, who indicated that the decrease of qP resulted in the decrease of Y(II). This might be caused by rate-limiting processes, which involves the complex processes of PSI and cytochrome b6/f^30^. In addition, Yu and Ong^31^ found that Y(II) and qP in leaves were decreased under red or yellow light compared with that under blue light.

Photosystem I (PSI) and II (PSII) are pigment reaction centers located on thylakoid membrane and participate in photosynthetic light reactions in oxygen photosynthesis of plants^32^. PSII uses the electrons produced by dissolving water as a source of its electron transport chain (including the electron-trons transferred on the PSI), ultimately reducing NADP+.

A higher Fv/Fm shading variation may result in lower photosystem I (PSI) fluorescence contribution due to changes in the distribution of light absorption between two lights. However, it may affect the light-protection response. When the balance between PSI and PSII is broken and more PSI is favored, it may affect the photoprotection response, such as the increase of NPQ and the decrease of qP, which is faster, indicating that Shading(B) may be caused by the overreduction of the receptors of the PSI system in the leaves under Shading(W) processing. It can also affect the photosynthetic performance of leaves^33^.

### Effects of different shading materials on yield and quality

Yield is a very important indicator in the evaluation of production practices. Under the same extreme low light intensity, different light quality environments had no significant difference in yield and yield components, indicating the rice yield reduction (low spikelet filling and reduction of the 1000-grain weight) was mainly due to the weakening of light intensity.

Under the same light intensity, the photosynthetic rate of barley seedlings grown under blue light was significantly higher than that under red and blue light^34^, which increased the proportion of blue light and significantly increased the protein content in plants^35^.

In this study, under shading treatment of different materials, the yield decreased, but the difference between the two treatments was not significant. The content of amylose in seeds under shade treatment was significantly lower than that in the control group; the chalkiness degree and rate was significantly higher than that in the control group, and the grain quality deteriorated, in contrast with Mo *et al*.^36^, who showed a significant reduction in chalkiness rate under black shading conditions during the whole period of grain filling.

Chalky formation of grains can be attributed to several factors, mainly owing to the influence of starch synthesis, which may affect the formation of starch grains. During the filling process, amylose content began to change after 15 d of shading. As time went on, the gap gradually widened, and the content of amylose under Shading (B) was higher than that under Shading (W). This is similar to the change of Chlb content in flag leaves. It is speculated that the change in chlorophyll in the leaves leads to the inhibition of the synthesis of photosynthetic products, and the corresponding changes in transportation to the grain, and finally directly affects the starch synthesis process in the grain, resulting in the reduction of amylose content.

The high proportion of blue light increases the actual photosynthetic efficiency, resulting in the photosynthetically active product under Shading (B) being higher than that under the Shading (W) and transported into the kernel, synthesizing more amylose. It adjusts the proportion of starch in the whole grain, such as less amylopectin, which is a key factor in the formation of chalkiness in grain. We hypothesized that changes in light quality caused different conditions in the growth and development of plants.

## Conclusion

In our research, the temperature and light photorecorder were used to accurately record the changes in canopy microclimate in two different shading materials simulating cloudy conditions, and some key factors affecting rice yield and quality were determined. Because of the change in light quality, different light components changed, which affected photosynthetic performance and the synthesis and transport of photo-contracted compounds finally reducing the quality of the grains. It is indicated that the decrease in light intensity during rice filling stage is the direct cause of yield reduction, and the change in light quality may be a key factor causing the decrease in grain quality. It is recommended that future research uses white gauze as the test material in the actual shading experiment, which is closer to the real situation of cloudy day than black nylon net; that is, the light intensity is reduced, the R/FR is decreased a little, the radiation intensity of other visible light (blue and green) is not greatly increased, and the light composition is also close to the natural cloudy sky than black nylon net.

## Methods

### Material

Field experiments were conducted at the farm of the Sichuan Agricultural University in Wenjiang (30°43′N and 103°52′E), Sichuan Province, China, in 2018. The soil was a medium loam with 28.5 g kg^−1^ organic matter, 1.5 g kg^−1^ total N, 0.94 g kg^−1^ total P, 17.5 g kg^−1^ total K, 137.2 mg kg^−1^ alkali hydrolysable N, 27.1 mg kg^−1^ Olsen-P, and 143 mg kg^−1^ exchangeable K in 2018. Four mid-late *indica* hybrid rice (*Oryza* sativa L.) varieties bred by Sichuan Agricultural University were used in the study: Huanghuazhan, Duohui I, Shuhui498, and Guichao II (conventional rice). Two different shading materials, white cotton yarn (Shading (W), Woven from pure cotton) and black nylon net (Shading(B), By nylon plastic warp and weft cross woven), were used for shading during the rice filling period, with 53% reduction of full natural light.

### Plant growth parameters

A two-factor, randomized block experiment with three replicates was conducted to investigate the effect of rice variety (4 landrace varieties) and shading stress (no shading control (CK) and 53% shading of two materials: white cotton yarn (Shading (W) and black nylon net (Shading (B)). For the shading treatment, rice plants were covered with shading materials from heading (August 1, 2018) to 30 d after heading (August 30, 2018). The screens were 2 m high and sufficiently large to maintain good ventilation and prevent lateral sunlight penetration. Six-week-old seedlings were transplanted at a spacing of 33.3 cm × 20.0 cm with two plants per hill in plots of 3.0 m × 10.0 m on May 23, 2018. Fertilization in all treatments consisted of 180 kg hm^−2^ of N as urea, 90 kg hm^−2^ of P2O5 as single superphosphate, and 180 kg hm^−2^ of K2O as potassium chloride. The high-efficiency irrigation technique described by Wang^31^ was used for water management.

### Sampling and measurements

At the heading stage, 300 similar-sized panicles per plot were labelled with small plastic tags. Shade measures were taken after flowering of rice. After 5, 10, 15, and 20 d of shading, chlorophyll in the leaf of the rice sword was extracted by acetone extraction method and the chlorophyll content was detected by spectrophotometer, as described in Wang^37^. Chlorophyll fluorescence of the flag leaf after 20 d of shading was determined using a MINI-PAM Photosynthesis Yield Analyzer (Heinz Walz GmbH, Germany). The measuring conditions and calculation methods have been described in Wang^37^ and Kalaji^38^. The leaves of rice sword were tested from 9:00 am to 11 am. When Fm (maximum dark adapted fluorescence), F_O_ (initial fluorescence) and Fv/Fm (maximum PSII photochemical quantum yield) were measured, the leaves were fully dark adapted for 20 min. Actinic light intensity is the light intensity when the actual photosynthetic efficiency of sword leaves is 0.5 in the natural light environment. 5–8 leaves were determined in each plot.

Microclimate instrument (179-THL; Apresys, China) was used to measure the microclimate environment (temperature and PAR (photosynthetically active radiation)) of the spike during the filling period. The spectral irradiance of different wavelengths in spike canopy during the filling period was measured by using a fibre-optic spectrometer (AvaSpec-2048; Avantes, Netherlands). Spectral irradiance was measured at wavelengths ranging from 400 nm to 800 nm at 0.6 nm intervals. The spectral irradiance was determined as the mean of different positions in crop canopies.

### Amylose content and appearance quality

Rice grains at 15, 20, and 30 d after shading and at harvest were used for the detection of amylose content, and the detection method was according to Zhou^39^.

Approximately 1 kg of hand-harvested grains and 100 labelled panicles were sampled at harvest from each plot in 2018. Grains were stored under ventilated conditions for 3 months for standby application, and then, they were shelled and milled. Chalky rice rate, which reflects the proportion of chalky grains to the total number of grains observed, was measured following the method of He^30,40^.

### Yield and yield components

At maturity, five plants from each plot were harvested and threshed to determine yield components such as grain filling degree and grain weight, while grain yield was recorded from a 7 m^2^ area in each plot by adjusting to a standard moisture content of 13.5%.

### Statistical analysis

ANOVA model was performed to test the effects of environment parameters in spike canopy during the filling period and the relationship of yield, yield components, amylose content, and appearance quality among different light conditions. The means of each treatment were compared by using the LSD-Fisher test at the 5% significance level. The correlation between phenotypes was calculated using the Person correlation model at the 5% significance level. All the analyses were run with SPSS v21.0 and Origin 9.0.
